# Evaluation of the Maxillary Sinus of Patients with Maxillary Posterior Implants: A CBCT Cross-Sectional Study

**DOI:** 10.3390/diagnostics12123169

**Published:** 2022-12-15

**Authors:** Lucas Lenyn Vieira Chaves, Lucas P. Lopes Rosado, Saulo Machado Piccolo, Liana Matos Ferreira, Kivanç Kamburoglu, Rafael Binato Junqueira, Maurício Augusto Aquino de Castro, Francielle Silestre Verner

**Affiliations:** 1 Department of Dentistry, Governador Valadares Campus, Federal University of Juiz de Fora, Governador Valadares 35010-180, Brazil; 2 Division of Oral Radiology, Department of Oral Diagnosis, Piracicaba Dental School, State University of Campinas, Piracicaba 13414-903, Brazil; 3 School of Dentistry of Ribeirão Preto, Department of Dental Materials and Prosthesis, University of São Paulo, Ribeirão Preto 14040-904, Brazil; 4 Independent Researcher, Vila Velha 29100-000, Brazil; 5 Departament of Dentomaxillofacial Radiology, Faculty of Dentistry, Ankara University, Ankar 06560, Turkey; 6 Faculty of Dentistry, Federal University of Minas Gerais, Belo Horizonte 31270-901, Brazil

**Keywords:** cone beam, computed tomography, dental implants, diagnostic imaging, maxillary sinus, maxillary sinusitis

## Abstract

Background: During oral rehabilitation, dental implants in the posterior maxilla can penetrate the maxillary sinus. The aim was to evaluate the presence of maxillary sinus abnormalities in patients with dental implants in the posterior maxillary region using cone-beam computed tomography (CBCT) images. Materials and Methods: This was a retrospective cross-sectional study, and CBCT scans of 199 patients (459 dental implants) were evaluated. Implants were assessed according to their relative location to the maxillary sinus floor (up to 2 mm from the maxillary sinus cortex, within 2 mm to intimate contact with the maxillary sinus cortex, apical third inside the maxillary sinus, two-thirds or more inside the maxillary sinus) and bone-fixation tissue (Alveolar ridge or Bone graft). Maxillary sinus abnormalities were classified. Kappa and Weighted Kappa and the Kruskal–Wallis test were applied. Results: A higher prevalence of mucosal thickening and non-specific opacification were observed in implants located within 2 mm to intimate contact with the cortex of the maxillary sinus floor. Of the 66 implants with apical thirds located inside the maxillary sinus, 31 (46.7%) were associated with sinus abnormalities and of all implants (*n* = 5) with two-thirds or more located inside the maxillary sinus, all of these were associated with sinus abnormalities. No association was observed in relation to implant bone-fixation tissue. Conclusions: This study found a significant association between dental implant placement near or within the sinus and sinus abnormalities, mainly mucosal thickening and non-specific opacification.

## 1. Introduction

Dental implants are widely used for posterior maxillary restorations. However, factors such as poor bone quality, extensive alveolar resorption, and maxillary sinus pneumatization following tooth loss can make this a challenging task [1]. Moreover, due to the anatomical proximity of the alveolar ridge and maxillary sinuses as well as vascularization and common innervations, the fixation of dental implants in the posterior maxillary region can trigger inflammatory processes in the maxillary sinuses, especially in cases where the integrity of the maxillary sinus floor is affected [2].

Accidental perforation of the maxillary sinus mucosa may lead to acute or chronic inflammation, and thickening of the sinus mucosa as well as the presence of mucous retention cysts or pseudocysts may increase the risk of ostium obstruction, eventually leading to sinusitis [3]. Given that approximately 40% of maxillary sinusitis cases are of odontogenic origin, possible dental causes must be included in the differential diagnosis and treatment of sinusitis [4]. 

While recent improvements in oral hygiene habits have led to a decline in periapical- and periodontal-disease-related odontogenic sinusitis, rates of sinusitis associated with iatrogenic etiology and/or surgical dental procedures in the posterior maxillary region have been increasing [5]. Inadequate treatment of sinusitis can lead to implant failure as well as more serious complications such as orbital cellulitis, optic neuritis, extradural and subdural infection, encephalitis, osteomyelitis, and, in rare cases, brain abscess [6]. Moreover, a study by Longhini et al. [6] suggested that an increase in sinusitis related to maxillary dental-implant surgery can be expected in the near future.

Preoperative diagnostics based solely on panoramic radiographs may not suffice to display anatomic variations of the maxillary sinus. Cone beam computed tomography (CBCT) is considered to be an effective method for analyzing sinus structure [7,8]. Existing cone-beam computed tomography (CBCT) images can be used to provide an accurate assessment of the relationship between sinusitis and the presence of posterior maxillary implants [3,5].

However, the majority of the literature addressing the connection between posterior maxillary implants and sinusitis consists of clinical case studies [2,5,9,10] and as a result, a full understanding of the role that the spatial relationship between maxillary implants and the maxillary sinus plays in sinus alterations has yet to be established. Therefore, the present study aimed to evaluate the correlations between maxillary sinus abnormalities and the presence of dental implants in the posterior maxillary region using existing CBCT images. The null hypothesis was that there was no correlation between maxillary sinus abnormalities and the presence of dental implants in the posterior maxillary region.

## 2. Materials and Methods

Existing CBCT images of 199 patients 111 female (mean age: 57.41 ± 10.30), 88 male (mean age: 60.09 ± 9.47), with a total of 459 dental implants taken between 2016–2018 were obtained from the database of a Dentomaxillofacial Radiology Clinic of a public university. A sample calculation was performed based on a pilot study; a significant difference between the results obtained can be observed. Images that included maxillary dentition to the full extension of the maxillary sinuses and at least one posterior maxillary (premolar/molar) dental implants were included. Images showing pathological alterations suggestive of cysts, tumors or fibro-osseous lesions in the maxillary sinus, maxillary fractures, or posterior teeth with periapical and/or periodontal lesions were excluded because of the association between inflammatory periapical lesions and sinus alterations [11]. All CBTC images were obtained using an I-Cat^®^ (Imaging Sciences International, Hatfield, PA, USA) CBCT device with the following acquisition protocol: 37.07 mAs, 120 kVp, field of view (FOV) 8 × 13 cm, 0.25 mm voxel and 26 s scan time with a 360° spin.

Evaluations were performed individually by 3 examiners, dental radiology specialists with experience in CBCT imaging, who were previously instructed on the proposed methodology and who participated in a pilot study comprised of 20 images that were not included in the final sample. Images were viewed on a 21.5 inch LCD monitor at a high resolution (1920 × 1080), Dell S2240L (Dell Computadores do Brazil Ltda., Eldorado do Sul, Rio Grande do Sul, Brazil) in a dimly lit room under standardized conditions. Images were evaluated dynamically using XoranCat^®^ software, version 3.0.34 (Xoran Technologies, Ann Arbor, MI, USA). In addition to coronal, sagittal and axial reconstructions, panoramic and oblique sections were available for viewing, and image enhancement tools (zoom, brightness, contrast) could be used at the examiner’s discretion. In order to avoid visual fatigue, images from a maximum of 20 subjects were evaluated per day. Intra-rater reliability was calculated by having each examiner re-evaluate 20% of the sample after 30 days.

It is worth mentioning that some implants fixed in bone graft did not show overextension into the maxillary sinus, that is, even if this bone graft was inside the maxillary sinus, some implants were restricted to the region of this bone graft. In these cases, these implants were not considered to be inside the maxillary sinus.

**Figure 1:**
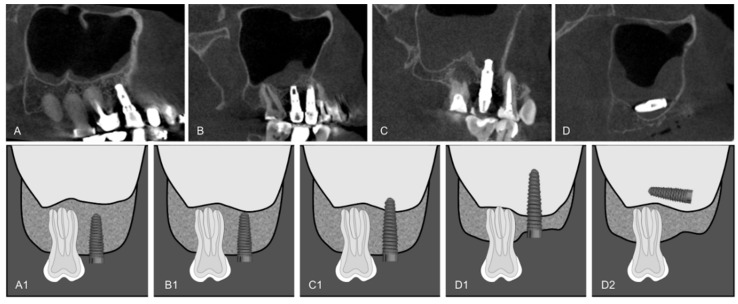
Dental implants in relation to the maxillary sinus: (**A**,**A1**) Up to 2 mm from the maxillary sinus cortex; (**B**,**B1**) Within 2 mm to intimate contact with the maxillary sinus cortex; (**C**,**C1**) Apical third inside the maxillary sinus; (**D**,**D1**) Two-thirds inside the maxillary sinus; (**D**,**D2**) Totally inside the maxillary sinus.

**Figure 2:**
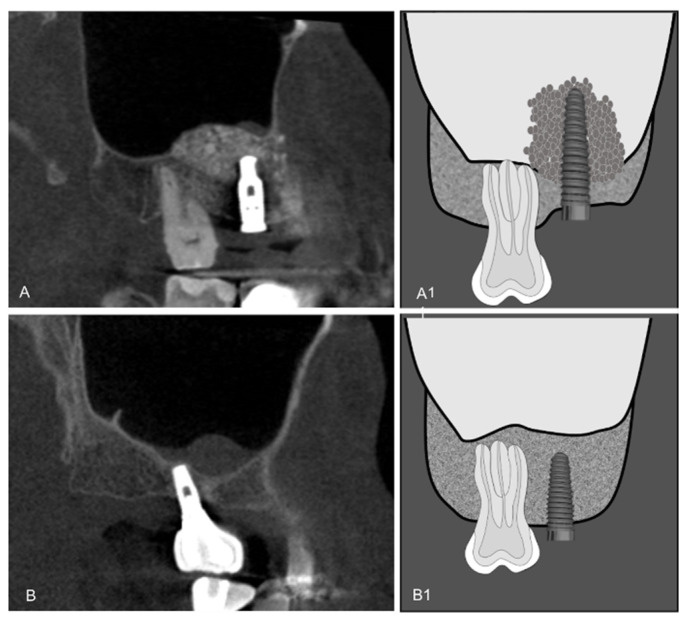
CBCT sagittal reconstruction and schematic drawing of: (**A**,**A1**) Dental implants fixed in bone graft and (**B**,**B1**) Dental implants fixed in alveolar ridge.

**Figure 3:**
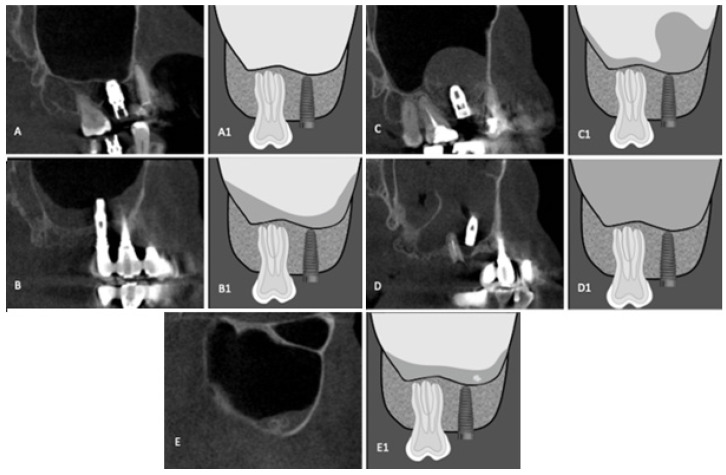
CBCT reconstructions and schematic drawing of maxillary sinus abnormalities. (**A**,**A1**) Normal Maxillary Sinus; (**B**,**B1**) Mucosal thickening; (**C**,**C1**) Antral pseudo cyst; (**D**,**D1**) Non-specific opacification; (**E**,**E1**) Antrolith.

Dental implants were classified by location in relation to the maxillary sinus (up to 2 mm from the maxillary sinus cortex; within 2 mm to intimate contact with the maxillary sinus cortex; apical third inside the maxillary sinus and two-thirds or more inside the maxillary sinus) (Figure 1), by bone-fixation tissue (bone graft and alveolar ridge) (Figure 2), and by maxillary sinus abnormalities (normal maxillary sinus; mucosal thickening; antral pseudo cyst; non-specific opacification and antrolith) (Figure 3). Maxillary sinuses were classified according to the absence/presence of alterations, using an adaptation of the classification proposed by Nunes et al. [11].

Kappa and Weighted Kappa values were used to evaluate intra- and inter-rater reliability. A descriptive analysis was performed, and the Kruskal–Wallis test was used to assess relationships between the studied variables. All statistical analysis was performed using SPSS Version 23.0 (SPSS Inc., Chicago, IL, USA), with the significance level set at 5%.

## 3. Results

Kappa and Weighted Kappa values varied between 0.89–0.94 for intra-rater reliability and 0.86–0.92 for inter-rater reliability, demonstrating almost perfect concordance. Therefore, the modes of the three evaluators’ responses were used in further evaluations. (Since no cases of sinus polyps were found, this classification will not be mentioned in the analysis).

Table 1 shows the distribution of implants by location in relation to the maxillary sinus, by bone-fixation tissue, and by maxillary sinus abnormalities according to insertion site. Out of a total of 459 implants, 155 (33.77%) were located at the maxillary first premolar (1PM) insertion site, 137 (29.85%) at the maxillary second premolar (2PM) site, 111 (24.18%) at the maxillary first molar (1M) site and 56 (12.20%) at the maxillary second molar (2M) site. The majority of the implants at the 2PM, 1M and 2M insertion sites were located within 2 mm of or in intimate contact with the cortex of the maxillary sinus floor (*p* = 0.000). For all sites, there was a higher prevalence of implants attached directly to the alveolar ridge as compared to bone graft (*p* = 0.01). It is noteworthy that 56.82% of the implants were not associated with alteration of the maxillary sinus, while mucosal thickening (30.29%) and nonspecific opacification (10.02%) were the most prevalent types of sinus alteration, followed by antral pseudocyst (1.97%) and antrolith (0.87%) (*p* = 0.708).

Table 2 shows the distribution of sinus alterations by implant location in relation to the maxillary sinus and by bone-fixation tissue. Higher prevalences of mucosal thickening and non-specific opacification were observed in implants located within 2 mm to intimate contact with the cortex of the maxillary sinus floor (*p* = 0.047). Of the 66 implants whose apical thirds were located inside the maxillary sinus, 31 (46.7%) were associated with sinus alterations, and while the number of implants had two-thirds or more located inside the maxillary sinus (*n* = 5), all of these were associated with sinus alterations. No association was observed between implant bone-fixation tissue and sinus alterations (*p* = 0.812).

Table 3 shows the distribution of implants according to their relationship with the maxillary sinus by bone-fixation tissue. No significant association was observed (*p* = 0.121).

## 4. Discussion

The absence of oral and maxillofacial function not only impairs chewing and phonation, it also results in maxillary and mandibular bone resorption The replacement of missing teeth has a significant functional and psychosocial impact, but also, dental implants placed in the alveolar bone are the therapy of choice for total or partial rehabilitation of edentulous patients [12,13]. However, implant rehabilitation in the posterior maxilla is hampered by Type IV bone—i.e., low-quality, low-density spongy bone with a very thin cortical layer—at insertion sites [14,15]. Furthermore, due to pneumatization of the maxillary sinus and/or resorption of the alveolar ridge, the region often presents limited bone height, and as a result, implants in these sites can penetrate the maxillary sinus and cause sinus alterations [2].

Numerous studies have shown that implant surgery can cause infectious diseases involving the oral and nasal cavities and maxillary sinuses [2,5,14,16,17,18]. Considering that approximately 10% of all cases of sinusitis are caused by odontogenic pathology, dental causes need to be considered in the diagnosis and treatment of sinusitis [5,16]. Implant-related paranasal sinusitis cases have increased as dental implantation has become the therapy of choice for the rehabilitation of edentulous areas [13], and while implant-related acute/chronic sinusitis and other maxillary complications have been described [13,18], their incidence and clinical relevance are not yet clearly understood [5,9,13,19,20].

Considering that the maxillary sinuses may appear within the imaging field of CBCT images of maxillary posterior teeth, it is important to have a good understanding of maxillary sinus anatomy and the clinical significance of anatomical variants [21,22,23,24,25,26,27]. Three-dimensional radiographic exams that provide axial-, coronal- and sagittal-plane imaging with good spatial resolution are better able to define the relationship between structures in this anatomically complex region, thus outperforming two-dimensional exams [21,22,24,28,29]. For this reason, the present study used CBCT for maxillary sinus analysis. It also assessed a much larger number of implants than previous studies [5,9,13,20,30].

Studies examining the connection between dental implants and sinusitis have used a variety of methodologies and suggested classifications for sinus alterations. Some previous studies were not inclusive in their definition of sinusitis, while others identified the origins of sinusitis, but not the specific type [28]. In the present study, maxillary sinus evaluation was based on a retrospective analysis of tomography images according to Nunes et al. [11], whose classification encompasses a broadly inclusive definition of sinus alterations. However, since posterior teeth with periapical lesions were excluded from the present study because of the association between inflammatory periapical lesions and sinus alterations [9,26,27], in contrast to Nunes et al. [11] periostitis was not included.

The mean age of participants was 57.41 for females and 60.09 for males. (This study did not analyze data by race). Considering that the number of lost teeth increases with age [31], the relatively high age of the study population may be due to the shift away from a predominantly curative approach to the current philosophy of prevention-based, minimally invasive dental treatment [32].

First molars accounted for a considerably higher proportion of the insertion sites (24.18%) than second molars (12.20%). This finding was expected, since the maxillary first molar, as the first permanent dental element to erupt [33], is exposed to risk factors such as caries for a longer time than other permanent teeth, making it more likely to suffer from tissue damage leading to tooth loss.

With regard to the high prevalence of implants in the premolar region (first premolars: 33.77%; second premolars: 29.85%), this may be due to aesthetic and psychosocial factors that motivate patients to seek rehabilitative treatment—as the extent of the smile extends to the premolar region in the majority of the population. Dental agenesis, which often affects the second premolars, may also contribute to the high rate of implants in this region [34].

Regarding the proximity of implants to the maxillary sinus, whereas the molar and second premolar implant sites showed the majority of implant apexes to be located within 2 mm of the maxillary sinus floor or in intimate contact with the maxillary sinus floor cortex, most of the majority of implants at premolar sites appeared to be at least 2 mm away from the maxillary sinus floor (Table 1). These findings are in line with previous studies and can be explained by the relatively smaller amount of bone available at molar and second premolar insertion sites and the greater proximity of these sites to the maxillary sinus floor. For example, Maillet et al. [28] found that sinusitis was more likely to be associated with the molars and Shahbazian et al. [27] found 54% of first molars examined to be in intimate contact with the maxillary sinus, as compared to only 38% of second molars, this may suggest that the apical portion of implants installed at these sites may also be in close proximity to the sinus floor.

Regardless of the insertion site examined, the present study found the prevalence of implants attached directly to the alveolar ridge to be much higher when compared to implants installed in bone graft, which accounted for less than 10% of all implants. This may be due to the limited implementation of grafting procedures, even when indicated.

The connection between exposure or displacement of dental implants in the maxillary sinus and sinusitis has not yet been fully elucidated. Reports suggest that the penetration of an implant into the sinus or nasal cavity may lead to sinusitis or rhinosinusitis, but that if the implant is covered by normal mucosa, sinus alterations may be prevented [2,18]. Jung et al. [9] in their experiments with dogs, observed that when implants penetrated the maxillary sinus floor less than 2 mm, they were spontaneously covered by healing mucosa. In contrast, a case study reported that the apexes of implants that penetrated the sinus floor by more than 4 mm remained exposed [2]. In line with these earlier studies, the present study found maxillary sinus alterations suggestive of mucosal inflammation in more than half of all cases in which implants were located inside the maxillary sinus (*n* = 36), regardless of the extent of penetration. Moreover, all cases in which two-thirds or more of an implant had penetrated the sinus (*n* = 5) were associated with sinus alterations.

Of the 459 implants evaluated, CBCT image analysis showed 198 (43.13%) to present sinus alterations. Among the sinus alterations detected, mucosal thickening (30.28%) and non-specific opacification (10.02%) were the most prevalent (40.30%), regardless of the implant insertion site. While some previous studies observed a higher prevalence of mucosal thickening (more than 50%) [2,9,30], Troeztchech et al. [30] reported a lower prevalence (33%).

The high rate at which mucosal thickening occurred when implants were located in close proximity to the sinus floor cortex suggests that this type of sinus alteration may occur as a tissue response to bone stimulation rather than to direct aggression to the lining by the implant. However, the risk of mucosal thickening may increase when implants are located within the sinus itself—as indicated by the high rates of tissue response to implants with the apical third or two-thirds located inside the sinus (23/66 and 3/5, respectively). Some authors have stated that mucosal thickening and other symptoms of odontogenic sinusitis may be delayed for some time, or may not be present, even in cases where sinus floor perforation has occurred [2,9,19].

## 5. Conclusions

A significant association between dental implant placement near or within the sinus and sinus alterations was found, mainly mucosal thickening and non-specific opacification. As a limitation, given the methodology of the present study, it was not possible to determine if mucosal thickening was present before implant placement. Due to the inherent limitations associated with retrospective cross-sectional studies, longitudinal studies that track the development of sinus alterations in implants placed within the maxillary sinus are recommended. Additionally, the results of the present study could support further studies and provide benefits to patient care.

## Figures and Tables

**Table 1:** Distribution of implants by insertion site, according to relationship to the maxillary sinus, bone-fixation tissue and sinus abnormalities.

	1PM ^a^	2PM ^b^	1M ^c^	2M ^d^	Total	*p* Value
**Relationship to maxillary sinus**						
Up to 2 mm from the maxillary sinus cortex	81 (17.647%)	34 (7.407%)	21 (4.575%)	7 (1.525%)	143 (31.154%)	<0.0001
Within 2 mm to intimate contact with the maxillary sinus cortex	63 (13.725%)	83 (18.082%)	68 (14.814%)	31 (6.753%)	245 (53.376%)	
Apical third inside the maxillary sinus	11 (2.396%)	19 (4.139%)	20 (4.357%)	16 (3.485%)	66 (14.379%)	
Two-thirds or more inside the maxillary sinus	0	1 (0.217%)	2 (0.435%)	2 (0.435%)	5 (1.089%)	
**Bone-fixation tissue**						
Alveolar ridge	151 (32.897%)	126 (27.450%)	98 (21.350%)	48 (10.457%)	423 (92.156%)	0.01
Bone graft	4 (0.871%)	11 (2.396%)	13 (2.832%)	8 (1.742%)	36 (7.843%)	
**Maxillary sinus abnormalities**						
Normal MS	91 (19.825%)	82 (17.864%)	60 (13.071%)	28 (6.100%)	261 (56.862%)	0.708
Mucosal thickening	39 (8.496%)	41 (8.932%)	37 (8.061%)	22 (4.793%)	139 (30.283%)	
Antral pseudo cyst	2 (0.435%)	1 (0.217%)	5 (1.089%)	1 (0.217%)	9 (1.960%)	
Non-specific opacification	21 (4.575%)	12 (2.614%)	8 (1.742%)	5 (1.089%)	46 (10.021%)	
Antrolith	2 (0.435%)	1 (0.217%)	1 (0.217%)	0	4 (0.871%)	

*p* Value—Kruskal–Wallis test. ^a^—Maxillary First premolar. ^b^—Maxillary Second premolar. ^c^—Maxillary First Molar. ^d^—Maxillary Second Molar.

**Table 2:** Distribution of maxillary sinus abnormalities according to implant-sinus relationship and bone-fixation tissue.

Relationship to Maxillary Sinus	Normal MS	Mucosal Thickening	Antral Pseudo Cyst	Non-Specific Opacification	Antrolith	*p* Valor
Up to 2 mm from the maxillary sinus cortex	81 (17.647%)	44 (9.586%)	1 (0.217%)	14 (3.050%)	3 (0.653%)	0.047
Within 2 mm to intimate contact with the maxillary sinus cortex	145 (31.590%)	69 (15.032%)	7 (1.525%)	23 (5.010%)	1 (0.217%)	
Apical third inside the maxillary sinus	35 (7.625%)	23 (5.010%)	1 (0.217%)	7 (1.525%)	0	
Two-thirds or more inside the maxillary sinus	0	3 (0.653%)	0	2 (0.435%)	0	
**Bone fixation tissue**						
Alveolar ridge	241 (52.505%)	125 (27.233%)	9 (1.960%)	44 (9.586%)	4 (0.871%)	0.812
Bone graft	20 (4.357%)	14 (3.050%)	0	2 (0.435%)	0	

*p* Value—Kruskal–Wallis test.

**Table 3:** Distribution of implants by bone-fixation tissue and implant-maxillary sinus relationship.

Relationship to Maxillary Sinus	Alveolar Ridge	Bone Graft	Total	*p* Value
Up to 2 mm from the maxillary sinus cortex	130 (28.32%)	13 (2.832%)	143 (31.152%)	0.121
Within 2 mm to intimate contact with the maxillary sinus cortex	223 (48.583%)	22 (4.793%)	245 (53.376%)	
Apical third inside the maxillary sinus	65 (14.161%)	1 (0.217%)	66 (14.378%)	
Two-thirds or more inside the maxillary sinus	5 (1.089%)	0	5 (1.089%)	

*p* Value—Kruskal–Wallis test.

## Data Availability

Not applicable.
